# Measuring Diagnostic Quality: The Capacity of Routinely Collected Data and Applications to Chronic Respiratory Disease

**DOI:** 10.2147/POR.S430705

**Published:** 2025-01-16

**Authors:** Rachael Mountain, Timothy Gatheral, Patrick Haslam, Kelly Heys, Jo Knight

**Affiliations:** 1 Lancaster Medical School, Lancaster University, Lancaster, UK; 2 University Hospitals of Morecambe Bay NHS Foundation Trust, Westmorland General Hospital, Kendal, UK

## Background

The demand on diagnostic services in England has risen year-on-year. The National Health Service (NHS) has identified the need for radical investment and reform of diagnostic services, both to prevent missed or delayed diagnoses, and to transform the process itself that can be duplicative and inefficient. New service delivery recommendations emphasize virtual consultations and community diagnostics to relieve pressure on acute services, an issue only exacerbated by the COVID-19 pandemic. There is considerable focus on the robust diagnosis of respiratory disease, with targets to reduce variation in spirometry testing nationally through improved training of primary care personnel.

However, there is currently no widely accepted approach for evaluating diagnostic performance. Measuring diagnostic quality must consider more than whether the final diagnosis is correct, but also the efficiency, timeliness, and rigorousness of the process. There is opportunity for advanced analytic methods able to incorporate a spectrum of information and account for the dynamic nature of the diagnostic process. Such methods would require appropriate input data sources with clinically rich information and linkage across healthcare tiers.^1^

Routinely collected health data holds huge potential for clinical research. Each patient interaction presents an opportunity to improve services and standard of care through data-driven analytics. However, the nature of routine data can limit its applications in research. The availability and quality of all variables required for analysis must first be assessed to evaluate the capacity of the data in answering the intended research question.

Existing literature recognizes the challenges of using routine data for evaluating diagnostics due to incomplete and erroneous coding, with an emphasis on the critical role of physicians in influencing the quality of routine data for research purposes.^2^,^3^ This study aimed to extend the discussion of the suitability of routine data for measuring diagnostic quality by considering the problem in the context of a specific data source and disease area. We aimed to illustrate key barriers using electronic health records (EHRs) from the Morecambe Bay Community Data Warehouse (CDW) applied to chronic respiratory disease (CRD).

## Methods

This report retrospectively analyzed primary and secondary care data from the Morecambe Bay CDW, an SQL Server owned by the University Hospitals of Morecambe Bay NHS Foundation Trust. The CDW uses pseudonymized NHS Numbers to individually link healthcare data across the Morecambe Bay, north-west England, covering 3 hospitals, 32 general practices (GPs), and a population exceeding 360,000. Patient demographic information was obtained from GP records.

CRD diagnoses (asthma, chronic obstructive pulmonary disease [COPD], bronchiectasis, and interstitial lung disease [ILD]) were identified from GP records by the first recording of a relevant SNOMED diagnosis code between 01/07/18 and 30/06/23. Only patients aged ≥35 years at time of diagnosis and with ≥12 months of continuous medical records prior to diagnosis were considered. Patients with missing age or sex information were removed from analysis. If an individual had more than one CRD diagnosis, these were treated as distinct events.

For each qualifying diagnosis, we extracted potential events in the diagnostic pathway in the six-month period prior to diagnosis. By “diagnostic pathway”, we are referring to the chain of events leading up to diagnosis. We obtained information for: respiratory symptoms (cough, wheeze, dyspnea, sputum, chest pain, fatigue, and weight loss) recorded in GP records using SNOMED codes; diagnostic tests conducted in both primary and outpatient care settings, using SNOMED codes and procedure names, respectively; and secondary care utilization, specifically outpatient respiratory clinic attendance, inpatient admissions using ICD-10 codes, and emergency department visits with mention of respiratory problems.

Data were stratified by diagnosis and summary data was calculated for age (mean and standard deviation), sex (percentage male) and presence of symptoms, tests and care utilization by percentage of diagnoses.

## Results

A total of 5922 CRD diagnosis events (43.4% COPD, 32.9% asthma, 13.6% bronchiectasis, 10.1% ILD) from 5435 individuals were included in this study. Table 1 summarizes the data findings including stratification by diagnosis.

**Table 1:** Summary of Extracted Data Variables Related to the Diagnostic Process for CRD. All Figures are Percentages Unless Specified Otherwise

	All (n=5922)	COPD (n=2573)	Asthma (n=1949)	Bronchiectasis (n=803)	ILD (n=597)
*Demographics*					
Age (mean ± standard deviation)	65.4 ± 13.3	66.3 ± 12.1	59.4 ± 13.6	71.4 ± 11.2	72.5 ± 11.8
Male	49.9	50.3	44.3	51.2	64.2
*Symptom information*					
Dyspnea	36.1	38.4	31.1	38.9	39.4
Cough	22.2	22.4	21.5	27.0	16.9
Sputum	14.4	16.2	10.0	21.2	11.9
Wheeze	12.7	12.0	17.3	10.3	4.2
Other^a^	8.0	9.4	4.8	9.7	9.5
Symptom absence	17.4	17.6	18.3	16.9	14.1
None	47.5	47.0	49.9	44.0	46.2
*Selected diagnostic tests*					
Blood test	63.6	65.3	53.1	74.7	76.2
Chest x-ray	41.1	44.0	31.0	53.1	45.4
Spirometry^b^	36.1	41.3	36.5	26.8	24.3
Peak expiratory flow	34.4	35.0	34.9	27.8	39.5
Oximetry	32.2	31.5	28.3	39.0	39.2
Reversibility^b^	23.2	28.4	24.6	13.6	9.5
Other lung function	22.6	27.8	19.6	21.4	38.4
Other	4.0	2.8	2.2	9.5	7.7
None	17.1	15.0	25.2	10.1	8.5
*Secondary care utilization*					
Outpatient clinic	26.3	22.4	12.6	44.2	63.5
Inpatient admission	17.1	17.9	8.9	23.7	32.0
Emergency department	9.3	10.3	6.0	11.8	11.7
None	60.8	62.6	78.6	41.3	21.1

**Notes**: ^a^“Other” symptoms are chest pain, fatigue, and weight loss. ^b^Spirometry is any evidence of spirometry being carried out whereas reversibility is when the spirometry has been specified as post-bronchodilation.

**Abbreviations**: CDW, Community Data Warehouse; COPD, Chronic Obstructive Pulmonary Disease; CRD, Chronic Respiratory Disease; EHR, Electronic Health Record; GP, General Practice; ILD, Interstitial Lung Disease; NHS, National Health Service; NLP, Natural Language Processing; QOF Quality and Outcomes Framework.

### Symptom Recording

Almost half of diagnoses had no SNOMED-coded symptom information in the six months prior to diagnosis. Absence of symptoms cannot safely be inferred from absence of symptom recording. Other explanations include incomplete data recording (including symptom information stored in free text), symptoms were not discussed, or an incidental diagnosis occurred following an unlinked medical event. The explicit recording of symptom absence was low, only 17.4%.

Removing patients without symptom information recorded from further analysis could induce bias by excluding milder or asymptomatic cases. Less obvious bias may result from demographic variability due to differences in data recording by GP sites. When aggregated at GP-level, the percentage of diagnoses without symptom information ranged from 30.1% to 83.7%.

### Diagnostic Tests

At least one diagnostic test was identified for 82.9% of diagnoses, but issues arise with interpreting results. X-ray imaging was the second most common test ordered prior to CRD diagnosis with highest rates seen for bronchiectasis. However, scan imagery and associated reports are not available in the CDW to interpret results. Descriptive SNOMED codes such as “Chest x-ray abnormal” exist yet lack clinical detail and had only been used in 4.9% of chest x-rays.

Quality and Outcomes Framework guidance continues to recommend confirmation of COPD diagnosis by quality assured post-bronchodilator reversibility for patients able to take the test. There was evidence of reversibility testing for 28.4% of COPD diagnoses, yet only 50.3% of this group had numeric results for both pre- and post-bronchodilation stored under explicit and easily identifiable SNOMED codes (eg, “Forced expiratory volume in one second/forced vital capacity ratio before[after] bronchodilator”). In 23.0% of cases, numeric results were missing altogether, potentially recorded in free text.

There is a lack of data pertaining to the motivation for a diagnostic test. Blood tests were the most common test ordered, yet these are not specific to one disease, or even a small group of conditions, and could have been ordered for purposes other than CRD diagnosis.

### Forming Care Pathways

We present evidence of care across the healthcare tiers. In total, 39.2% of diagnostic pathways included secondary care services with higher percentages observed for rarer diseases. However, the CDW does not contain free text fields, referral letters, or clinician reports. Without a connecting narrative, there is uncertainty in which healthcare events are part of the same chain of care and have contributed toward a diagnosis, similar to the issue of motivation behind diagnostic tests.

### Suspected Cases

Our analysis only included cases that result in diagnosis. However, we can infer that identifying suspected cases (where a patient is suspected of having a disease but is not diagnosed, either because the diagnosis was ruled out or the patient was not adequately followed up) presents a challenge. There are SNOMED codes for “Suspected asthma” and “Suspected COPD”, but these had only been used in 13.6% and 6.4% of asthma and COPD diagnoses respectively. No equivalent codes exist for the rarer diseases, bronchiectasis and ILD. Other variables could act as proxies, including diagnostic tests, symptoms, referrals, or a combination, but there are other conditions for which such events could apply.

## Discussion

This report has evaluated and illustrated the capacity of routinely collected health data for measuring diagnostic quality using examples from the Morecambe Bay CDW and CRD. Routine health data hold huge potential to provide feedback for transforming diagnostic services to meet increasing demand and improve standard of care. However, by exploring data quality in a specific setting, we have identified data-level barriers that must first be addressed to assess diagnostic performance. The novelty of this work lies in the in-depth study based on a specific set of phenotypes and the specificity of considering diagnostic accuracy. However, these issues also generalize beyond the scope of this project and can be broadly grouped into two themes: data recording practices and data access barriers.

### Data Recording

Data with rich clinical information such as GP observations will be essential to understanding diagnostic quality.^1^ However, our findings support previous literature that there are fundamental problems at the data recording level.^2–4^ Without consistent and high-quality data recording, both over time and between GPs, we are unable to distinguish incomplete data collection from incomplete diagnostic pathways. Standardizing data recording practices in primary care will be paramount to facilitating high-quality evidence-based health services research and could theoretically be implemented through national and international disease guidelines. We present specific recommendations regarding standardization.

First, standardization is needed in terms of the information to be recorded for each diagnosis. The consideration of symptoms is a likely first event in a diagnostic pathway, yet our results show this information is substantially under-recorded in primary care, particularly symptom absence. Other potentially key information, including commentary on scan imagery and codes indicating suspected disease, face similar barriers of inconsistent usage. This standardization will reduce the significant levels of missingness currently present in health data.

Second, standardization regarding the specific SNOMED codes used. SNOMED is the most comprehensive clinical terminology product globally and a critical tool for research with primary care EHRs. However, the hierarchy of the coding system creates multiple ways of recording similar information. The use of ambiguous codes can lead to misclassification by a researcher, or the information being missed altogether,^4^ as illustrated by post-bronchodilation spirometry results.

### Data Access

Access to individually linked data will be crucial to the task of measuring diagnostic quality.^1^ Health data research in England is moving in the direction of wider access to individually linked data with recommendations outlined in the Goldacre Review and the recent funding of NHS Secure Data Environments to centralize health data sub-nationally. This study supports existing recommendations by demonstrating the proportion of diagnostic pathways that traverse healthcare tiers. The fragmentation of data across different health systems is an established barrier to research with routine data and prevents a full picture of patients’ healthcare journeys.^5^

Unstructured data, including free text fields, narrative reports, and referral letters, is needed to improve clarity and completion of the diagnostic pathway, yet access to unstructured data is often limited to researchers in accordance with information governance. We recommend the further implementation of methods for drawing structured data from free text, such as natural language processing (NLP). For example, Bean et al use the open-source tools CogStack and MedCAT to undertake annotation of the entire text content of the Electronic Health Record from King’s College Hospital, London, UK. They produced a dataset covering 9 years that contains 157M SNOMED concepts generated from 9.5M documents for 1.07M patients. This demonstrates that NLP could be used both to withdraw information from historical data and to supplement gaps in structured data, including clinical motivation and narrative to link events, as well as symptom and diagnostic tests information not stored under SNOMED concepts. Additionally, NLP implemented in current EHR systems could support real-time coding of structured data to improve usage of correct clinical coding.^2^,^6^

## Limitations

In this study, diagnosis events were identified by the first recording of a relevant SNOMED diagnosis code. This method was selected based on previous validation studies for identifying CRD patients from EHRs, yet these studies consider only whether the patient has the disease in question, and not the precise time of diagnosis. Patients can be treated for a condition before a diagnosis code is recorded. Alternatively, a code may be recorded when it is in fact only a working diagnosis, influenced by financial incentives such as the QOF in England. Suspected disease SNOMED codes do not qualify for QOF patient registers, a possible explanation for their low usage in our data. This source of uncertainty is a limitation of our study and since we examine the six-month period prior to diagnosis, our results may change under different definitions of time of diagnosis. However, it is also an issue beyond the scope of this study and the problem links into the data recording theme. Established coding practices combined with validation studies are required for accurately identifying time of diagnosis in EHRs.^7^

Other limitations include that the data explorations have been kept brief and more patterns in the data could be uncovered by exploring relationships with, for example, age, sex, and time, as well as practice variation. Second, we used a 5-year study period which covers the COVID-19 pandemic, a time of significant disruption to healthcare services. However, both pre- and post-pandemic data are included to minimize bias. Finally, we have focused on a specific case study and have not explored the generalizability of our results to other routine data sources or disease areas.

## Conclusion

Measuring diagnostic quality using routinely collected data will require improvements in data recording and data access. A standardization of data recording practices in primary care is needed to promote consistent, high-quality, and easily interpretable data. However, even with perfect coding, structured EHRs leave gaps in the diagnostic pathway and fail to capture contextualization and the integration of multiple diagnostic parameters by the physician. Unstructured data present in healthcare documents combined with NLP methodology may provide solutions.

Until the steps required to make accurate and timely diagnosis are adequately recorded in EHRs, routine data will be unable to measure and evaluate diagnostic quality. Future research must first focus on implementing and evaluating the recommendations outlined in this study before application of methodology such as machine learning to address the question of measuring diagnostic quality using routine data.

## Data Availability

CDW data are not publicly available for patient confidentiality reasons. SNOMED and ICD-10 codes used for analysis are available at: https://doi.org/10.17635/lancaster/researchdata/651.
